# Effect of neoadjuvant iodine-125 brachytherapy upon resection of glioma

**DOI:** 10.1186/s12885-022-09504-5

**Published:** 2022-04-12

**Authors:** Congxiao Wang, Chao Liu, Jun Chen, Han Jiang, Wei Zhang, Lili Yang, Xueda Li, Zixiang Li, Lijing Peng, Xiaokun Hu, Peng Sun

**Affiliations:** 1 Department of Interventional Medical Center, the Affiliated Hospital of Qingdao University, No. 1677 Wutaishan Road, Shandong 266000 Qingdao, China; 2 Department of Radiology, Pingyi People’s Hospital, 273300 Linyi, Shandong China; 3 Department of Neurosurgery, Fenyang hospital of Shanxi, 032299 lvliang, Shanxi China; 4 Qingdao Medical College of Qingdao University, 266000 Qingdao, Shandong China; 5 Department of Clinical Laboratory, the Affiliated Hospital of Qingdao University, Qingdao, China; 6 Department of Neurosurgery, the Affiliated Hospital of Qingdao University, No. 1677 Wutaishan Road, Shandong 266000 Qingdao, China

**Keywords:** Neoadjuvant ^125^I brachytherapy, Glioma, Surgical resection, Combination therapy, Tumor shrinkage

## Abstract

**Background:**

A more extensive surgical resection of glioma contributes to improved overall survival (OS) and progression-free survival (PFS). However, some patients miss the chance of surgical resection when the tumor involves critical structures.

**Purpose:**

The present study aimed to assess the feasibility of neoadjuvant ^125^I brachytherapy followed by total gross resection for initially inoperable glioma.

**Methods:**

Six patients diagnosed with inoperable glioma due to invasion of eloquent areas, bihemispheric diffusion, or large tumor volume received ^125^I brachytherapy. Surgical resection was performed when the tumor shrank, allowing a safe resection, assessed by the neurosurgeons. Patients were followed up after surgery.

**Results:**

Shrinkage of the tumor after adjuvant ^125^I brachytherapy enabled a total gross resection of all six patients. Four patients were still alive at the last follow-up, with the longest survival time of more than 50 months, two of which returned to everyday life with a KPS of 100. Another two patients had neurological injuries with KPSs of 80 and 50, respectively. One patient with grade II glioma died 34 months, and another with grade IV glioma died 40 months after the combined therapy.

**Conclusions:**

In the present study, the results demonstrated that ^125^I brachytherapy enabled a complete resection of patients with initially unresectable gliomas. ^125^I brachytherapy may offer a proper neoadjuvant therapy method for glioma.

## Background

Gliomas originate from glial, stem, or neuronal precursor cells. According to histology, World Health Organization (WHO) classified glioma into four grades (grade I-IV). Gliomas are now better characterized by molecular changes. Usually, grade I to II gliomas are low-grade gliomas, and grade III and IV gliomas are called high-grade or malignant gliomas [1]. For glioma, surgical resection was the first choice [1, 2]. Numerous studies have suggested the therapeutic efficacy of maximal surgical resection while avoiding neurological damage to improve progression-free survival (PFS) and overall survival (OS) [3]. However, due to the situation of eloquent areas, bihemispheric diffusion, or considerable volume limitation, the extent of resection (EOR) is limited [1, 4]. Recent studies suggested that preoperative chemotherapy could decrease the tumor volume, thereby facilitating a safe and extensive resection [5, 6] and prolonging the PFS and OS of patients with gliomas.

Gliomas easily recur due to their characteristic diffuse infiltrative spread from the origin site [7, 8]. Based on these characteristics, local treatment with ^125^I is possible. Brachytherapy with iodine-125 seeds offered a safe and effective local treatment option for patients, including glioma in the eloquent brain [9–12]. Furthermore, ^125^I brachytherapy showed advantages in tumor shrinkage as the ^125^I seeds were inserted into the tumors, continuously releasing γ rays. However, the feasibility of the therapy strategy, combining neoadjuvant ^125^I brachytherapy with subsequent surgical resection, has never been thoroughly evaluated to the best of our knowledge. Thus, we undertook the present study to assess whether neoadjuvant ^125^I brachytherapy could facilitate a safe total gross resection for patients with initially unresectable gliomas and whether the combination therapy is effective.

## Methods

 All methods were performed in accordance with the relevant guidelines and regulations.

### Patient criteria

Patient data were obtained from the hospital database, spanning from January 01, 2016, to December 31, 2017. The retrospective study was approved by the Institutional Review Board of the local hospital (Reference number: QYFY WZ LL 26,579), and the requirement for informed consent was waived. The inclusion criteria were as follows: (1) Age ≥ 18 years old; (2) Enhanced MRI or CT deemed unresectable gliomas upon neurosurgical evaluation; (3) Combined therapeutic approach, consisting of neoadjuvant ^125^I brachytherapy and subsequent surgical resection; (4) Availability of adequate laboratory examination information, including hematologic parameters, clotting, hepatic and renal function, etc. Patients with glioma involving the brain stem or ependymal surface were excluded.

### Iodine-125 implantation

Iodine-125 seeds were implanted, as we previously reported [12]. Briefly, patients were safely fixed on the CT bed with a negative pressure vacuum pad. Then, ^125^I seeds (diameter of 0.8 mm, length of 4.5 mm, seeds radioactivity: 0.7 mCi; half-life of 59.4 days; Model 7711, Beijing Atom and High Technique Industries, Inc., Beijing, China) were implanted according to the preimplantation plan made with the computerized treatment planning system (TPS; Beijing Astro Technology Ltd. Co., Beijing, China). Briefly, Urethral catheterization was performed after general anesthesia. A homemade locator was placed on the head to confirm the puncture point. Holes were made with an electric cranial drill, flat needles were inserted into the tumors, and ^125^I seeds were implanted with the needles. Dynamic CT scans were performed during the surgery to confirm the distribution of the seeds. The average number of seeds implanted into the six patients was 55 (range, 21–100). Patients were required to stay in bed for 24 h, and dehydration medications were given for 7–14 days. After the implantation of the ^125^I seeds, patients received outpatient examinations every two months. The neurosurgeons evaluated the examinations. Surgical resection was conducted when the glioma shrank to a suitable size.

## Results

**Fig. 1:**
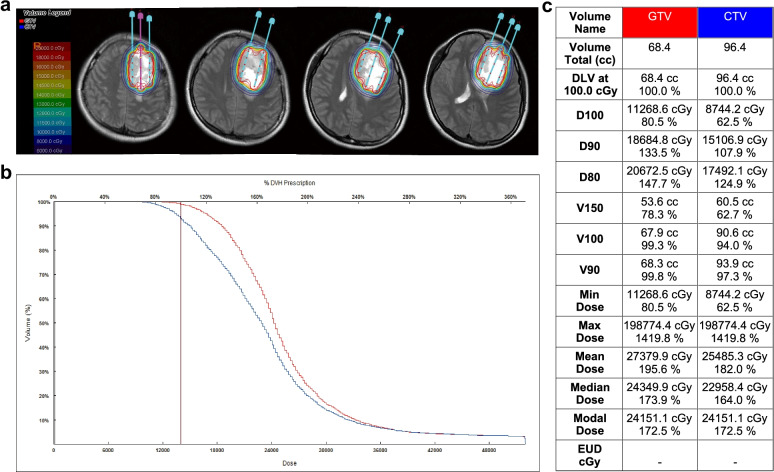
Pre-implantation plan of ^125^I brachytherapy for a patient (Figs. 1, 2, 3 and 4 are from the same patient) with grade IV glioma. **a** The gross target volume (GTV) was outlined with a red line, the clinical target volume (CTV) was outlined with a blue line. Different colored lines showed the dose distribution. Additionally, the needle paths were designed with TPS. **b** Pre-implantation dose-volume histogram (DVH) of GTV and CTV. **c** Pre-implantation D100, D90, D80, V150, V100, V90 of the GTV and CTV

**Fig. 2:**
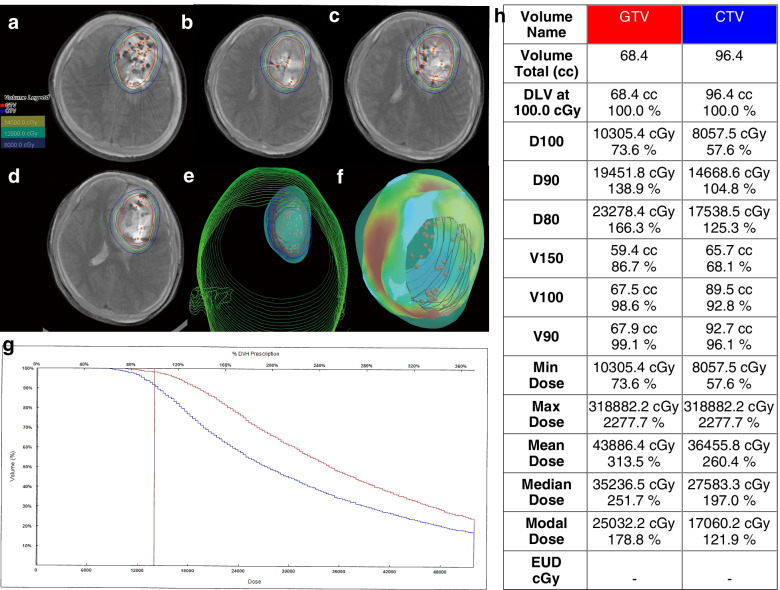
Post-implantation verification of ^125^I brachytherapy for a patient with grade IV glioma.** a-d** The gross target volume (GTV) was outlined with a red line, the clinical target volume (CTV) was outlined with a blue line. Different colored lines showed the dose distribution after ^125^I implantation. **e**-**f** 3D-reconstruction of the CT-MRI infusion images after ^125^I implantation. **g** Post-implantation DVH of GTV and CTV. **h** Post-implantation D100, D90, D80, V150, V100, V90 of the GTV and CTV

As Tables 1 and 2 show, two females and four males were included in this study. The median age was 43.5 years old (range 22–65). The median follow-up time from diagnosis was 32.5 months (range 21–47). The average number of seeds implanted into the six patients was 55 (range, 21–100). The median prescribed dose was 110 Gy (range, 90–140 Gy). The presenting symptoms were headache in three patients, nausea in one patient, vomiting in one patient, dizziness in one patient, myodynamia decline in one patient, vision decline in one patient, language disorders in two patients, and seizures in five patients. The median KPS was 85 (range 70–90). The anatomical location of the tumor was frontal in four patients, parietal in one patient, and left hippocampus in one patient. Three patients had low-grade gliomas, and the other three had high-grade gliomas. One patient had her first neuropathological diagnosis by biopsy; the other five were diagnosed with craniotomy. One patient with recurrent glioma had previously received surgical resection accompanied by external beam radiotherapy (total dose 50 Gy, 2 Gy/time) and chemotherapy with temozolomide (TMZ, 75 mg/m2). One patient had previously received bevacizumab treatment (400 mg/time, 4 times total). The other four patients received ^125^I brachytherapy as initial therapy. ^125^I seeds were implanted with TPS guidance, and verification of the dosage distribution was conducted immediately after implantation (Figs. 1 and 2). All patients received total surgical resection after ^125^I brachytherapy with a median therapy interval of 6 months (range 2–23 months).

**Table 1:** Patient characteristics

Characteristics	*N* = 6
**Age (yrs.)**	
Median (Range)	43.5 (24–65)
**Sex**	
Male	4
Female	2
**KPS**	
Median (Range)	85 (70–90)
> 80	3
≤ 80	3
**No. of seeds**	
Average (Range)	55 (21–100)
**Extent of Resection postbrachytherapy**	
Gross total resection	6
Partial resection	0
Biopsy	0
**EBRT Pre-125I**	
Yes	1
No	5
**Adjuvant chemotherapy-Pre**	/
Yes	2
No	4
**Tumor position**	
Frontal lobe	4
Occipital lobe	0
Parietal lobe	1
Temporal lobe	0
Hippocampus	1
**Tumor location**	
Left hemisphere	3
Right hemisphere	3
Both hemisphere	0
**Tumor Diameter**	
≥5 cm	2
<5 cm	3
**Symptoms**	
Headache	3
Nausea	1
Vomit	1
Dizziness	1
Myodynamia decline	1
Vision decline	1
Language disorders	2
Seizures	5
**Histology**	
Low-grade	3
High-grade	3
**Tumor Type**	
Recurrent	2
Primary	4
**No. of seeds**	
Median (Range)	65(21–113)
**PD (Gy)**	110(90–140)

*EBRT *External beam radiotherapy, *PD *Prescribed dose

**Table 2:** Details of the six patients during the therapy

No. of Patient	Sex	Age	KPS	Symptoms	Previous therapy	No. of 125I seeds implantation	PD	Anatomical location	Grade of gliomas	Subsequent surgery	KPS (last follow up)	Survival time (months)
1	Female	24	80	Headache, vision decline, seizures	NO	80	140	Frontal (large volume)	WHO IV	Total gross resection	100	51
2	Female	52	90	Dizziness, seizures	Surgical resection + EBRT + Temozolomide	50	90	Frontal (eloquent area)	WHO II	Total gross resection	0	34
3	Male	41	90	Myodynamia decline, seizures	Bevacizumab	53	130	Parietal (bihemispheric diffusion)	WHO IV	Total gross resection	0	40
4	Male	46	80	Language disorders, seizures	NO	26	90	Left hippocampus	WHO I-II	Total gross resection	80	31
5	Male	22	90	Headache, seizures	NO	21	110	Frontal (large volume)	WHO II	Total gross resection	100	52
6	Male	65	80	Headache, vomit, Language disorders, seizures	NO	100	110	Frontal (eloquent area)	WHO III	Total gross resection	50	33

*KPS *Karnofsky Performance Scale, *PD *prescribed dose, *WHO *World Health Organization, *EBRT *external beam radiotherapy

**Fig. 3:**
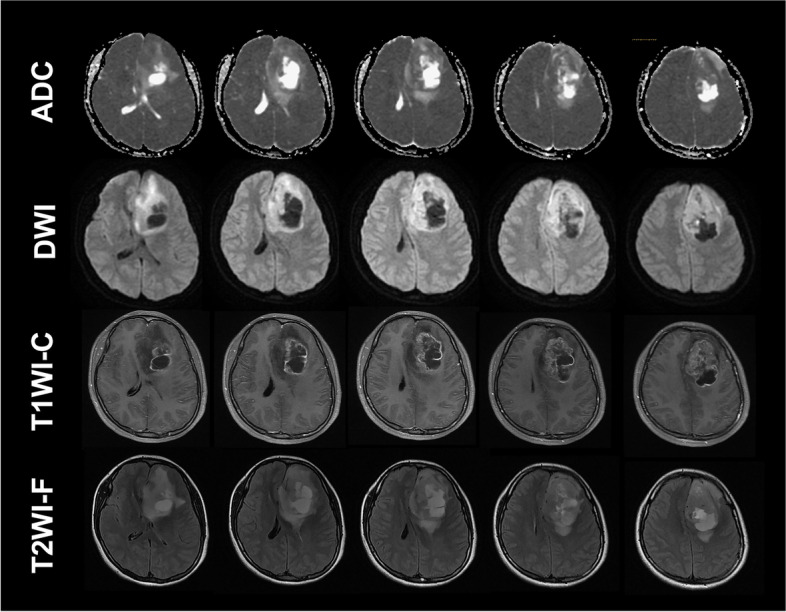
Brain MR images of the patients with grade IV glioma. Apparent diffusion coefficient (ADC), diffusion-weighted image (DWI), T1WI-contrast, and T2WI-flair images of the brain showed the occupation of the tumor and severe brain edema around the glioma was shown on the images

**Fig. 4:**
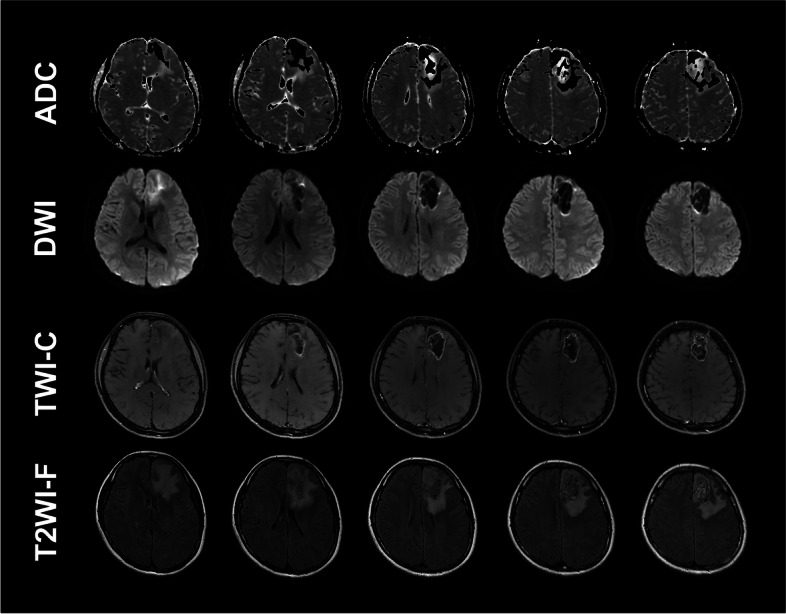
Brain MR images of the patients with grade IV glioma, eight months after the ^125^I implantation. ADC, DWI, T1WI-contrast, and T2WI-flair images showed the glioma shrink obviously after the ^125^I implantation. The brain edema also improved compared with the brain images eight months ago

**Fig. 5:**
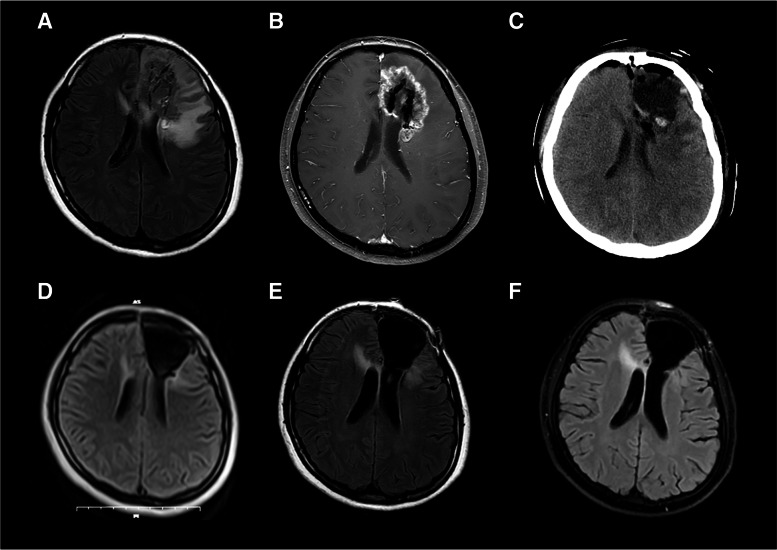
Routine imaging examinations of the patients. **A** T2WI-Flair image of the patient on 2018.07.27. **B** T1WI-contrast image of the patient on Dec.16, 2018. **C** CT image of the patient on January 27, 2019 after the surgical resection. **D** T2WI-Flair image of the patient on Apr.22, 2019. **E** T2WI-Flair image of the patient on May 08, 2019. **F** T2WI-Flair image of the patient on June 23, 2019

One patient with glioblastoma returned to everyday life with a KPS of 100 and was still alive at the last follow-up (survival time, 51 months) (Figs. 3, 4 and 5). Another patient with grade II oligodendroglioma had already survived for 52 months at the last follow-up, with a KPS of 100, and was still alive when we followed up. No neurological symptoms were found in these two patients after the combination therapy. They were free from seizures after the combined therapy. Another two patients with grade III and II gliomas were alive when we followed up (survival time: 33 months and 31 months, respectively), with KPSs of 50 and 80, respectively. One patient (grade II glioma) died with a survival time of 34 months. Furthermore, another patient with glioblastoma died with a survival time of 40 months.

## Discussion

In the present study, we retrospectively analyzed patients with gliomas who did not qualify for surgical resection. ^125^I brachytherapy was used as adjuvant treatment for surgery. After significant tumor shrinkage due to 125I implantation, gross total resection was achieved in six cases, and the patients had a long survival time.

According to the European guidelines [2], surgical resection is the first-line treatment for gliomas, aiming to perform maximal tumor mass resection and avoid long-term neurological deficits. However, gliomas located in the eloquent cortex, deep structure, and corpus callosum or large volumes limit the extent of surgical resection, sometimes causing severe neurological deficits. Neoadjuvant therapy has been widely used in tumor downstaging, enabling secondary resectability [13]. Moreover, neoadjuvant therapy has been successfully used in glioma therapy [5, 6, 14]. ^125^I brachytherapy is emerging as a new therapeutic approach for tumors [15–19] and has also been used in glioma therapy, primarily as salvage therapy (Table 3). ^125^I brachytherapy showed effective local control of the gliomas. However, whether ^125^I brachytherapy can be used as a neoadjuvant therapy approach for patients with unresectable gliomas has not been explored.

**Table 3:** Summary of major published studies of ^125^I brachytherapy for gliomas

Study	No. of patients	Type of gliomas	Implanted dose (Gy)	Median survival
B. Suchorska et al. [20]	99(primary) 73(recurrence)	Primary and recurrence anaplastic glioma WHOIII	60(primary), 60(recurrence)	28.9 months(primary), 21.4 months (recurrence)
David A. Larson et al. [21]	38	Progressive or recurrent glioblastoma multiforme	300 (at 5-mm depth)	52 weeks
Philipp Kickingereder et al. [22]	103 (primary) 98(recurrence)	Primary and recurrent glioblastoma	60	11.1 months(primary) 10.4(recurrence)
Patrick Y.Wen et al. [23]	56	Glioblastoma	50	22 months (reoperation) 13 months (no further surgery)
Berstein et al. [24]	23	Malignant glioma	60–70	14 months

In the present study, six patients received ^125^I brachytherapy. Surgical resection was not suitable for these patients due to the location and volume of the tumors. Two patients received other therapies before, and four received ^125^I brachytherapy as initial therapy. Most patients showed noticeable tumor shrinkage 2–8 months after ^125^I brachytherapy regardless of the tumor type. After a complete evaluation by the neurosurgeon, the six patients received total surgical resection of tumors at different time points after ^125^I brachytherapy. One patient with glioblastoma returned to everyday life after the combination therapy with a KPS of 100. No neurological system disorders were found after the therapy. Another patient with grade II oligodendroglioma also achieved a KPS of 100 after therapy and was free from seizures.

Furthermore, the patient was ready for marriage at the last follow-up. The two patients discussed above all faced a large tumor volume before the surgery. After therapy with ^125^I, the tumor volume decreased, which enabled total surgical resection. All six patients received the combination therapy of ^125^I brachytherapy followed by total surgical resection. Four of them were still alive when we last followed up, with a survival time of more than 31 months. Only one patient had language disorders after the therapy. Moreover, all the patients who survived were free from seizures. Two patients died with a survival time of more than 34 months.

The results showed that ^125^I brachytherapy showed practical efficacy in tumor shrinkage, enabling a subsequent total surgical resection. No severe neurological damage was found after the combined therapy. Furthermore, patients were free from seizures after the therapy. All patients survived a long time after therapy. For recurrent or primary gliomas, the combination therapy showed apparent efficacy. However, only six patients were included in this study, including low- and high-grade gliomas. A large number of cases should be included in further studies.

## Conclusions

The results were not statistically significant. However, the results were positive and indicated a promising therapeutic approach for gliomas. Thus, we believe that neoadjuvant ^125^I brachytherapy followed by surgical resection might provide opportunities to patients with gliomas.

## Data Availability

The datasets used and/or analyzed during the current study are available from the corresponding author on reasonable request. The data of the patients are available from the electronic medical record of the Affiliated Hospital of Qingdao University.

## Abbreviations

CT: Computed Tomography
EOR: Extent of Resection
HCC: hepatocellular carcinoma
KPS: Karnofsky Performance Status
MRI: Magnetic Resonance Imaging
OS: Overall Survival
PFS: Progression-free survival
TMZ: Temozolomide
TPS: Treatment Planning System
WHO: World Health Organization

## Declarations

(Reference number: QYFY WZ LL 26579). The study was performed in accordance with the Declaration of Helsinki.

Fenyang hospital of Shanxi waived the need of informed consent, and Dr. Jun Chen got the approval and consent to participate section in the manuscript from Fenyang hospital of Shanxi .
